# Regenerative Capacity of Old Muscle Stem Cells Declines without Significant Accumulation of DNA Damage

**DOI:** 10.1371/journal.pone.0063528

**Published:** 2013-05-21

**Authors:** Wendy Cousin, Michelle Liane Ho, Rajiv Desai, Andrea Tham, Robert Yuzen Chen, Sunny Kung, Christian Elabd, Irina M. Conboy

**Affiliations:** Department of Bioengineering and QB3 Institute, University of California, Berkeley, California, United States of America; University of North Dakota, United States of America

## Abstract

The performance of adult stem cells is crucial for tissue homeostasis but their regenerative capacity declines with age, leading to failure of multiple organs. In skeletal muscle this failure is manifested by the loss of functional tissue, the accumulation of fibrosis, and reduced satellite cell-mediated myogenesis in response to injury. While recent studies have shown that changes in the composition of the satellite cell niche are at least in part responsible for the impaired function observed with aging, little is known about the effects of aging on the intrinsic properties of satellite cells. For instance, their ability to repair DNA damage and the effects of a potential accumulation of DNA double strand breaks (DSBs) on their regenerative performance remain unclear. This work demonstrates that old muscle stem cells display no significant accumulation of DNA DSBs when compared to those of young, as assayed after cell isolation and in tissue sections, either in uninjured muscle or at multiple time points after injury. Additionally, there is no significant difference in the expression of DNA DSB repair proteins or globally assayed DNA damage response genes, suggesting that not only DNA DSBs, but also other types of DNA damage, do not significantly mark aged muscle stem cells. Satellite cells from DNA DSB-repair-deficient SCID mice do have an unsurprisingly higher level of innate DNA DSBs and a weakened recovery from gamma-radiation-induced DNA damage. Interestingly, they are as myogenic *in vitro* and *in vivo* as satellite cells from young wild type mice, suggesting that the inefficiency in DNA DSB repair does not directly correlate with the ability to regenerate muscle after injury. Overall, our findings suggest that a DNA DSB-repair deficiency is unlikely to be a key factor in the decline in muscle regeneration observed upon aging.

## Introduction

Adult organisms are subject to various physical and biochemical injuries throughout their lifespan and regenerative capacities differ greatly across organs. In vertebrates, skeletal muscle robustly regenerates during youth and adulthood owing to muscle stem cells, known as satellite cells. In uninjured muscle, satellite cells reside in a quiescent state between the plasma membrane (sarcolemma) and the basal lamina of the mature muscle fiber [1]–[3].

Muscle injury provides a synchronizing signal for the activation/proliferation of satellite cells and similarly, satellite cell isolation from uninjured muscle mimics an injury causing their simultaneous activation *ex vivo* [3], [4]. The majority of these activated satellite cells differentiate into myoblasts, migrate to the injury site, and then either differentiate into new, multi-nucleated myotubes or fuse with existing ones to repair the injury [5]. A smaller portion of these cells return to quiescence to maintain the stem cell pool [2].

Upon aging, skeletal muscle regenerative capacity drastically declines as manifested by a loss of functional tissue, accumulation of fibrotic tissue, and reduced satellite cell-mediated myogenesis in response to injury [3], [6]–[8]. While alterations of extracellular signals have been linked to age-related decline in satellite cell performance [3], little is known about their intrinsic properties such as their ability to repair DNA damage and how any changes could affect muscle regeneration. Such an understanding would be important in order to combat muscle aging and its growing impact on the elderly. Perturbations in the DNA DSB repair response mechanisms of other organ stem cells have been shown to alter stem cell function, which potentially promotes aging [9]–[11]. In addition, it has been demonstrated that hematopoietic stem cells accumulate DNA DSB with age, which contributes to their impaired function [12], [13].

The different types of DNA damage such as base modifications, nucleotide mismatches, or single and double strand breaks can occur both by endogenous (cellular metabolism, replication errors) as well as exogenous (ionizing radiation, mechanical stress, chemical exposure) sources. The most dramatic type of DNA lesion is through DNA double strand breaks (DSBs) that may result in genome instability and cell death, thus promoting tumorigenesis and/or premature aging [14], [15].

In an effort to respond to the drastic nature of DNA DSBs, a variety of signaling cascades are activated, leading to DNA repair, cell cycle arrest, and/or apoptosis. In eukaryotes, DNA DSBs are primarily repaired through error-prone non-homologous end-joining (NHEJ), which rejoins DNA ends with little or no sequence homology and which occurs mainly during G0-G1 and early S phases [16], or error-free homologous recombination (HR), which uses the sister chromatid as a template to match and seal broken ends and is restricted to late S-G2 phase.

Multiple factors coordinate DNA DSB repair in both pathways, starting with the recognition of DNA DSB sites by the protein complex Mre11-Rad50-Nbs1, and subsequent recruitment and phosphorylation of ATM and H2AX [17]. ATM-dependent phosphorylation of the histone protein H2AX generates *γ*-H2AX and is considered to be the first event in the cellular protein recruitment process following DNA damage. *γ*-H2AX plays a central role in DNA DSB signaling and repair, since it is used as a platform to recruit other DNA repair proteins to the sites of DNA DSBs. NHEJ is executed by recognition, recruitment, and binding of the DNA-dependent protein kinase (DNAPK) complex to the site, which in concert with the Artemis protein, stimulates the processing of the DNA ends to be transformed to 5′-phosphorylated ligatable ends [1]–[3], [18], [19]. There are two main complexes: DNAPK and X4-L4. The active serine-threonine kinase DNAPK complex is composed of proteins Ku70, Ku80, and DNAPK catalytic subunit (DNAPKcs). This complex stimulates the processing of the DNA to complete NHEJ by the X4-L4 protein complex, composed of Ligase IV, XRCC4, and XLF that ultimately ligates the broken DNA [3], [4], [20]–[22].

Alternatively, HR requires processing of the DNA DSB into a single stranded DNA (ssDNA) 3′-overhang in order to utilize the genetic information from an undamaged homologous DNA sequence. The newly-formed ssDNA overhangs are rapidly bound by replication protein A, followed by the formation of nucleoprotein filament structures composed of Rad51 multimers and other associated HR proteins, such as Rad52 and BRCA1 [5], [23], [24]. This newly formed nucleoprotein filament locates a homologous sequence on the undamaged sister chromatid DNA and initiates DNA strand exchange. DNA polymerase and Ligase I then extend and ligate the DNA to complete the HR process [2], [17].

The goal of this study was to determine whether an accumulation of DNA damage plays a major role in the age-specific decline of myogenicity. In order to assess the presence of DNA DSBs in muscle stem cells and the ability of those cells to repair DNA damage, we compared the number of *γ*-H2AX foci and the expression of key DNA repair machinery proteins, as well as the expression of DNA damage response genes between young and old satellite cells. As a positive control for deficient DNA repair, we used severe combined immunodeficiency (SCID) mice, a common model of DNA DSB repair deficiency [3], [6]–[8], [25]–[27]. These mice have a major DNA DSB repair defect and display hypersensitivity to ionizing radiation due to a hypomorphic point mutation in the DNAPKcs gene, leading to a decrease in DNA-PK activity [3], [28]–[30].

In the present study we observed no significant accumulation of innate DNA DSBs in muscle stem cells with age. Moreover, upon muscle injury, old satellite cells –known to have an impaired regenerative response– did not show any difference in the number of DNA DSBs, the expression of key DNA DSB repair proteins and DNA damage response genes, and their radiosensitivity. Interestingly, muscle stem cells from DNA-DSB-deficient SCID mice were as capable as those of young wild type mice to regenerate muscle and to form myogenic colonies despite an accumulation of DNA damage and a pronounced radiosensitivity, thereby suggesting that the inability to repair DNA DSBs does not correlate with the ability to regenerate muscle after injury. Overall, our findings suggest that a DNA DSB repair deficiency is not a major contributor to the decline in muscle regeneration seen upon aging.

## Results

### No difference in DNA DSBs in muscle stem cells with aging

**Figure 1:**
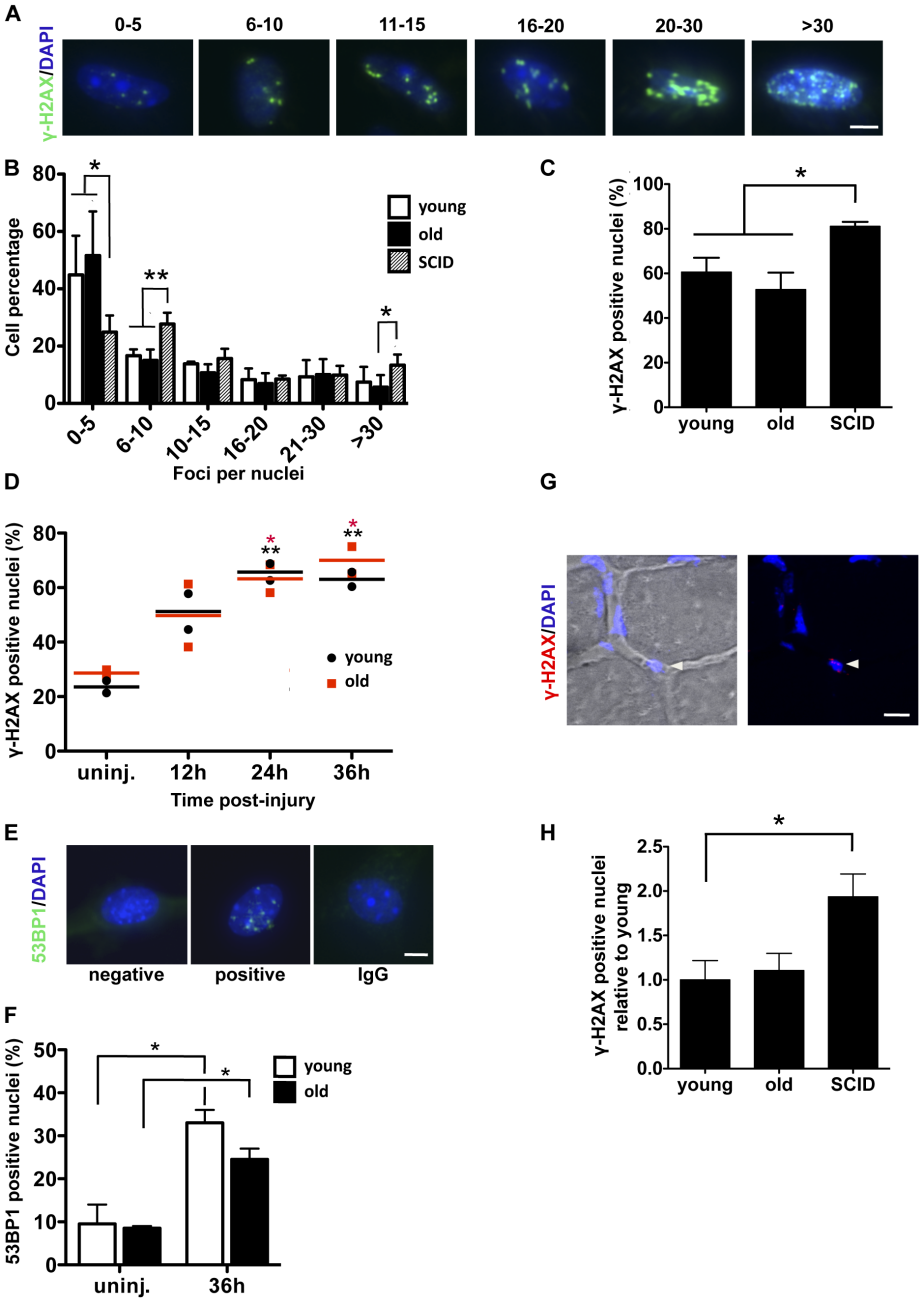
No difference in DNA DSBs in muscle stem cells with aging. (A-F) Satellite cells from uninjured muscle or activated by injury for different times were isolated from hind leg muscle of young and old C57BL/6 mice and young SCID mice and were immunostained for γ-H2AX (green) (A-D) or for 53BP1 (green) (E-F), and counterstained with DAPI (blue). (A) Representative pictures of binned ranges of γ-H2AX foci/nuclei. Scale bar represents 5 µm. (B-C) Quantification of γ-H2AX foci in activated satellite cells 72 hours post muscle injury. At least 140 cells were counted per mouse. Data represent the mean +/- SEM, n = 4 mice per group; two-tailed unpaired Student’s t- test, *: p value < 0.05. (B) Binned ranges of foci/nuclei. (C) Percentage of γ-H2AX-positive cells ( = 5 foci/nuclei). (D) Quantification of γ-H2AX positive nuclei in satellite cells from uninjured muscle (uninj) and injured muscle 12, 24, and 36 hours post injury. Young and old mice are symbolized by black dots and red squares, respectively. Each dot or square represents one animal and the lines correspond to the mean. 2 young and 2 old mice were used per time point and at least 100 cells were counted per mouse, two-tailed unpaired Student’s t-test, *: p value < 0.05, **: p value < 0.01 (black * young uninj. vs 12, 24 or 36 hours, red * old uninj. vs 12, 24 or 36 hours). (E) Representative picture of 53BP1 positive cell. Scale bar represents 5 µm. (F) 53BP1 positive cell numbers ( = 5 foci/nuclei) were compared between young and old mice from uninjured muscle and 36 hours after injury. At least 100 cells were counted per mouse. Data are presented as mean +/- SEM, n = 2 mice per condition, two-tailed unpaired Student's t-test, *: p value < 0.05 (uninjured vs. injured), no age-specific difference was observed. (G-H) Tibialis anterior muscles of young and old wild-type mice as well as young SCID mice were cryosectioned. 10 ?m cryosections were immunostained for γ-H2AX and counterstained with DAPI. Confocal Z-series images were projected into a single plan using maximum pixel intensity. (G) Magnification of a representative picture, showing a γ-H2AX positive satellite cell (white arrowhead). DAPI is represented in blue and γ-H2AX is represented in red. Scale bar represents 10 µm. (H) The percentages of ?-H2AX positive cells in satellite positions relative to young were compared between young, old, and SCID mice. At least 150 nuclei were scored per mouse on Z-stack projected images. Data represent the mean +/- SEM, n = 3 mice per group; two-tailed unpaired Student’s t- test, *: p value < 0.05.

Previous reports have shown that satellite cells, which undergo rapid activation and proliferation within 48–72 hours after muscle injury in young mice, exhibit a remarkable age-specific decline in performance, as observed by diminished proliferation and poor myogenicity when isolated from aged animals [3], [6], [9]–[11], [31], [32]. In order to test whether or not DNA DSBs contribute to impaired satellite cell function with age, *in vivo* activated satellite cells were isolated from cardiotoxin (CTX)-injured muscle of 2 to 4 month-old C57BL/6 mice (referred to henceforth as “young mice”) and of 20 to 24 month-old C57BL/6 mice (referred to henceforth as “old mice”) 72 hours post injury, and were quantified for the number of *γ*-H2AX nuclear foci, which is a commonly used method to monitor DNA DSB induction and repair [12], [13], [33], [34]. As shown in **Figure 1A**, *γ*-H2AX foci/DSBs were clearly detected in activated satellite cells. However, no age-specific differences in the numbers of *γ*-H2AX foci were observed when counted either as the percentage of total *γ*-H2AX positive cells or cell counts that are binned into 0–5, 6–10, etc. foci per nuclei (**Fig. 1B, C**). Activated satellite cells from young and old mice were isolated for these studies with a high purity as demonstrated by the high percentage of cells positive for Myf-5 (87% in young, 80% in old) or for MyoD (77% in young, 72% in old) (**Fig. S1A, B**). Furthermore, activated satellite cells from young SCID B6.CB17-Prkdcscid/SzJ mice (referred to henceforth as “SCID mice”), which have a defect in DNA DSB repair [14], [15], [28], showed a higher percentage of *γ*-H2AX positive nuclei (81%±4) than did satellite cells isolated from both young and old mice (61%±13 and 53%±15, respectively). When binned, satellite cells isolated from SCID mice had a greater percentage of cells within the higher ranges of foci (6–10 and >30 foci) and a significantly lower percentage of cells within the lower range of *γ*-H2AX foci (0–5 foci) (**Fig. 1B**).

Even though no difference in DNA DSBs was observed between satellite cells from young and old mice 72 hours after activation by muscle injury, it is possible that a difference existed at earlier time points, causing DNA DSB-induced cell cycle arrest or cell death, and thereby eliminating these cells from the assays conducted at 72 hours. To address this, we performed a kinetic experiment. *γ*-H2AX positive nuclei were scored in satellite cells harvested from young and old uninjured muscles and from young and old muscle after 12, 24, and 36 hours of *in vivo* activation by muscle injury. As shown in **Figure 1D**, satellite cells from uninjured muscle (where most satellite cells are quiescent) had low percentages of *γ*-H2AX positive cells (24% in young, 29% in old) when compared to activated satellite cells. This percentage increased as activation and proliferation occured and reached a plateau by 24 hours. In satellite cells isolated 12 hours post injury, we observed a noticeable, but not statistically significant increase in DNA DSBs as compared to satellite cells from uninjured muscle. At 24 and 36 hours, satellite cells are proliferating and the number of DNA DSB-positive cells significantly increases and reaches 63–70% (Fig. 1 D), which is comparable to the level observed at 72 hours (**Fig. 1C**). However, no age-related difference in the percent of *γ*-H2AX positive nuclei in satellite cells was detected in either uninjured or injured muscle at any time point (**Fig. 1C, D**).

Although *γ*-H2AX is commonly used to monitor DNA DSBs, recent studies have shown that *γ*-H2AX foci formation may not be exclusive to DNA DSBs and may also reflect other DNA lesions such as single strand breaks at stalled replication forks [16], [35]. Therefore, to confirm that the *γ*-H2AX foci increase at 72 hours post injury reflected an increase in DNA DSBs, we co-stained the highly proliferative activated by injury satellite cells for *γ*-H2AX and 53BP1 (which is involved in DNA repair through NHEJ). Co-localization analysis of *γ*-H2AX and 53BP1 foci corroborated the conclusion that that the *γ*-H2AX positive foci being studied reflected DNA DSBs (**Fig. S1C**). We also quantified the percentage of 53BP1-positive satellite cells isolated from young and old uninjured muscle and from young and old muscle 36 hours post injury, when the level of *γ*-H2AX positive cells plateaus (**Fig. 1E, F**). There was a significant increase in the percentage of 53BP1 positive cells at 36 hours post injury as compared to that of uninjured muscle; however, similarly to the *γ*-H2AX data, no age-related difference in the percentage of 53BP1 positive nuclei was found between young and old satellite cells from both uninjured and injured muscle (**Fig. 1E, F**). These results confirm that there is no accumulation of DNA DSBs in satellite cells of old as compared to young mice, and that there is an age-independent increase in the number of DNA DSBs following muscle injury. Moreover, these data suggest that the NHEJ repair pathway is active in both young and old satellite cells.

To confirm the physiological *in vivo* significance of the data obtained from isolated satellite cells, muscle sections from young, old, and SCID mice were immunostained for *γ*-H2AX (**Fig. 1G**). We scored the number of *γ*-H2AX positive cells in satellite cell positions and found, in good correlation with the *ex vivo* data, no age-specific increase in *γ*-H2AX positive quiescent satellite cells (**Fig. 1H**). Significantly more *γ*-H2AX foci in sublaminar satellite cells were detected in young SCID mice than in young C57BL/6 mice, providing a good internal control to these results (**Fig. 1H**).

### Assessment of satellite cell radiosensitivity and DNA DSB repair pathways with age

**Figure 2:**
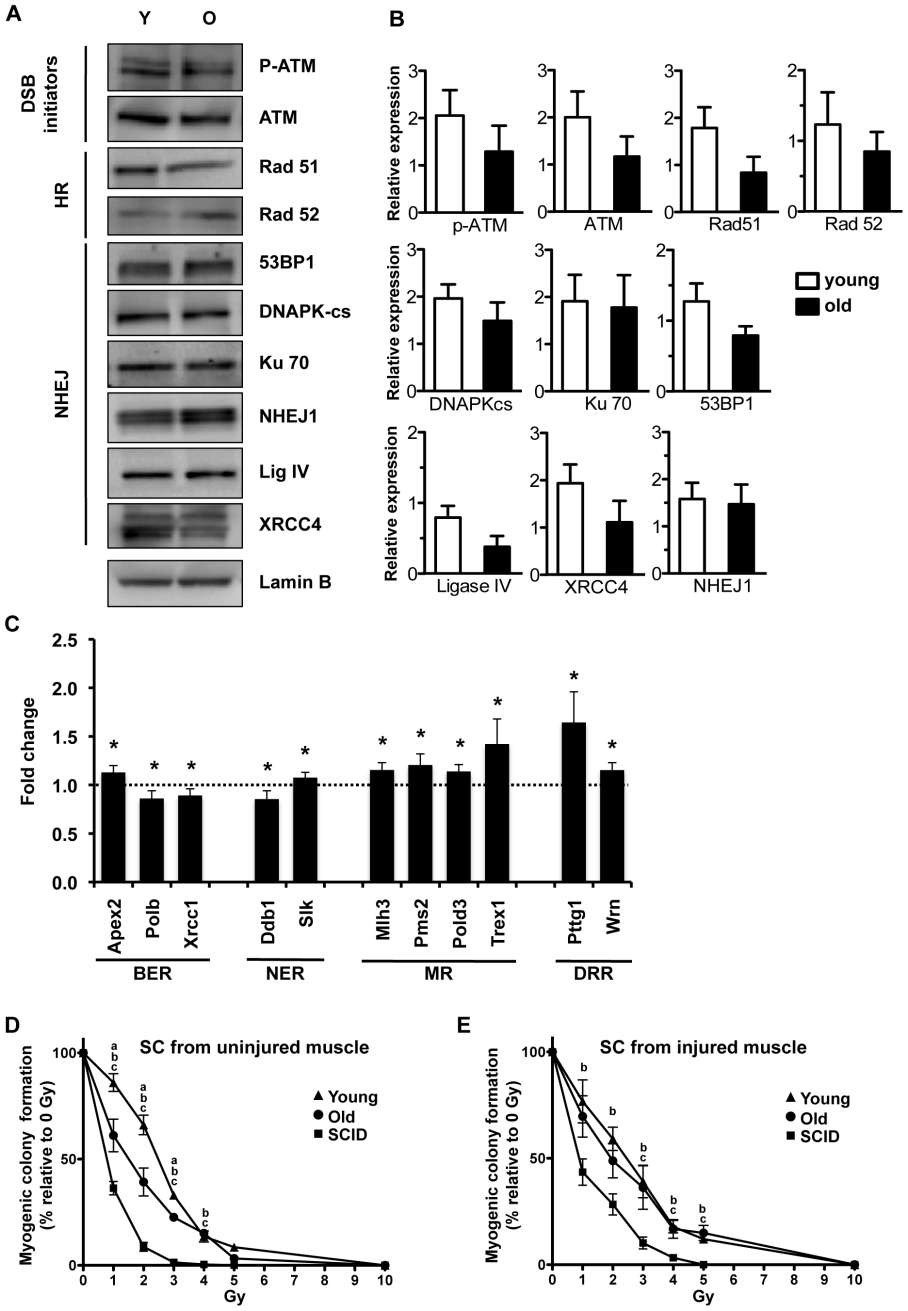
Assessment of satellite cell radiosensitivity and DNA repair pathways with age. (A–B) The expression of major proteins involved in homologous recombination and non-homologous end joining were detected using western blotting. Protein nuclear extracts were prepared from activated satellite cells 72 hours post muscle injury. Lamin B was used as an internal loading control. (A) Representative images of western blots. (B) Quantification using Image J software. Data represent the mean +/− SEM, n = 3 to 5 (n = a pool of muscles from 3 mice), two-tailed unpaired Student's t-test, no significant difference. (C) Graphical representation of data from **Table S1**. Gene expression of old relative to young satellite cells are presented as fold change with a 95 percent confidence interval for the 12 genes with a statistically significant relative expression, n = 3 mice per group, two-tailed unpaired Student's t-test, *: *p* value<0.05. The dotted line represents the young level. BER: Base Excision Repair, NER: Nucleotide-Excision Repair; MR: Mismatch Repair, DRR: DNA Repair Related. (D–E) Satellite cells were freshly isolated from uninjured (D) or 72 hours after injury (E) muscle of C57BL/6 young and old mice, and of young SCID mice. Cells were plated at low density, irradiated at indicated Gray (Gy) doses, and cultured for 10 days. Myogenic colonies formed by irradiated cells were quantified and represented relative to their respective non-irradiated controls. On average, 178, 77, and 228 myogenic colonies were scored per mouse for young, old, and SCID uninjured non-irradiated respectively and 67, 34, and 91 myogenic colonies were scored per mouse for young, old, and SCID injured non-irradiated respectively. Data represent the mean +/− SEM, n = 3 to 4 mice per group, two-tailed unpaired Student's t-test, a, b or c: *p* value<0.05, (a: young vs old, b: young vs SCID, c: old versus SCID).

In order to better characterize satellite cells' ability to repair DNA damage with age, we quantified the expression levels of DNA DSB response proteins using western blotting: p-ATM and ATM, DNA DSB initiators; 53BP1, DNPKcs, Ku70, XLF, LigIV, XRCC4, members of the NHEJ repair pathway; and Rad51 and Rad52, involved in HR. As shown in **Figures 2A and B**, a tendency for p-ATM, ATM, Rad51, Rad52, 53BP1, LigIV, and XRCC4 protein expression to decrease with age in activated satellite cells was observed. However, none of these differences were statistically significant.

Moreover, we assessed the global transcription of DNA damage response genes involved in DNA DSB repair as well as apoptosis, cell-cycle, base excision repair, DNA mismatch repair, etc. using quantitative RT-PCR. Similarly to our western-blot data, no clear age-specific difference was observed. Out of 122 transcripts, only 12 showed a significant difference of expression in old versus young activated satellite cells, but these showed only minor fold changes (0.85–1.2 for ten transcripts; 1.4–1.64 for two transcripts, n = 3; **Table S1,** **Fig. 2C**). The pituitary tumor transforming gene (pttg1) codes for a protein that acts at the interface between DNA repair and cell cycle arrest. Its expression displayed the highest fold change with a 1.64 fold increase in old as compared to young satellite cells. Interestingly, four members of the mismatch repair pathway group – Mlh3, Pms2, Pold3, and Trex1 – also showed a slightly increased expression in old as compared to young activated satellite cells (**Fig. 2C**). However, all the changes were less than 2-fold and none of the genes related to DNA DSB repair showed a significant age-related difference (**Table S1 and Fig. S2**). These data suggest that there may be a slight age-specific decline in the DNA repair efficiency of old satellite cells, as compared to young.

Based on our data, such a potential difference does not cause a significant age-specific change in the DSB foci number in non-activated and activated satellite cells under physiological conditions; however, it could manifest upon an induced DNA damage. Hence, we examined whether the recovery from radiation-induced damage is diminished in old satellite cells. To this end, we compared the radiosensitivity of satellite cells from young and old mice and from young SCID mice in both uninjured and injured muscle conditions. Cells were plated at clonal density in myogenic growth medium and were exposed to various levels of gamma-radiation from 0 to 10 Gray (Gy), which causes DNA DSBs. Ten days later, the number of myogenic colonies was quantified as percentages relative to the number of colonies formed by non-irradiated satellite cells (**Fig. 2D, E**). Myogenic colonies were identified by their morphology (**Fig. S3A, B**) as confirmed by the immuno-detection of Pax-7 and desmin (**Fig. S3C**). As shown in **Figure 2D**, there was a mild but statistically significant increase in the radiosensitivity of satellite cells from old uninjured muscle as compared to young at 1, 2, and 3 Gy of irradiation, which manifested as a decrease in myogenic colony formation by 25%, 26%, and 10%, respectively. Interestingly, the age-related increase in radiosensitivity of the wild type old satellite cells was not as drastic as that of SCID mice, which have a known defect in DNA DSB repair (**Fig. 2D**). Namely, at 2 Gy, only 9% of satellite cells from SCID mice were able to form myogenic colonies. At 4 Gy, satellite cells from young and old mice were equally sensitive to radiation, and both were significantly more resistant than were satellite cells from SCID mice. No age-specific difference in radiosensitivity was observed in satellite cells activated by injury (**Fig. 2E**). Activated satellite cells from young and old mice recovered from the irradiation equally as well and both recovered much better than activated satellite cells from SCID mice (**Fig. 2E**). Interestingly, satellite cells isolated from both young and old uninjured mice showed similar sensitivity to gamma-radiation with respect to their age-matched injured samples (**Fig. S4**).

These data confirm that young and old wild type satellite cells repair DNA DSB much better than cells from SCID mice and suggest that when DNA repair capacity is challenged to its limits by gamma-radiation, a slight age-specific decline is observed in satellite cells from old uninjured muscle.

### DNA DSB-repair-deficient SCID mice display robust muscle regeneration

**Figure 3:**
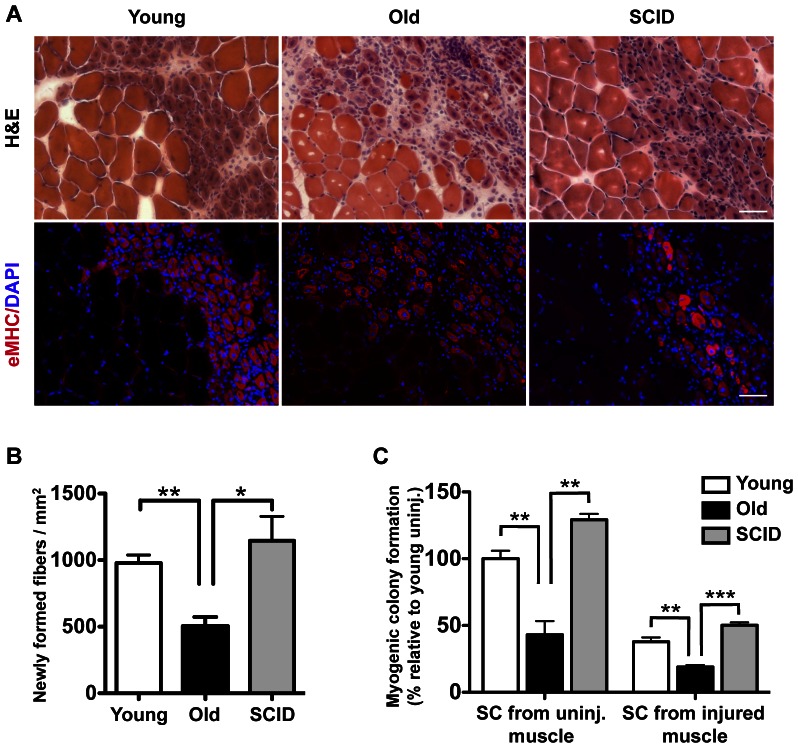
No decline in muscle regeneration and myogenic colony formation in DNA DSB repair-deficient SCID mice. (A) Tibialis anterior muscles of young and old wild type mice and young SCID mice were injured, and success in regeneration was assessed 5 days later. Histology was performed on cryosections using hematoxylin and eosin (H&E), and newly formed fibers were detected by eMHC immunofluorescence (red) and counterstained with DAPI (blue). Scale bar represents 50 µm. (B) Newly formed fibers per square millimeter were quantified using the H&E staining. Data represent the mean +/− SEM, n = 3 to 4 mice per group, two-tailed unpaired Student's t-test, *: *p* value<0.05; **: *p* value<0.01. (C) Satellite cells (SC) from uninjured (uninj.) or 72 hours after injury muscle were isolated from young and old wild type mice and young SCID mice and plated at low density. Myogenic colonies were quantified and represented relative to young uninjured. On average, 178, 77 and 228 myogenic colonies were scored per mouse for young, old and SCID uninjured respectively and 67, 34, and 91 myogenic colonies were scored per mouse for young, old, and SCID injured respectively. Data represent the mean +/− SEM, n = 3 to 4 mice per group, two-tailed unpaired Student's t-test, **: *p* value<0.01; ***: *p* value<0.001.

In order to evaluate whether an increase in satellite cell radiosensitivity correlates with an impaired function, we measured muscle regeneration *in vivo* in young, old, and SCID mice by assessing newly formed regenerative myofibers and the ability of satellite cells from those mice to form myogenic colonies. As previously reported [3], [6], [17], [31], [36], the efficiency of muscle regeneration decreased with age: the number of newly-formed eMHC positives myofibers at the injury site was significantly lower in old than in young mice and the typical age-specific scarring of the injured area was observed in old mice (**Fig. 3A, B**). While recovery from gamma-radiation was significantly diminished in satellite cells from SCID mice (**Fig. 2D, E**), muscle regeneration was as robust in SCID mice as in young mice and, indeed, much better than in old mice (**Fig. 3A, B**). To further investigate satellite cell function, we compared their ability to form myogenic colonies *in vitro* in the absence of gamma-radiation. Satellite cells isolated from old wild type mice displayed a defect in formation of myogenic colonies *in vitro*, reflecting the age-specific decline in myogenic proliferation observed in previous studies [7], [37]. In perfect correlation with the robust muscle repair, no difference was observed between satellite cells from SCID and young wild type mice in myogenic colony formation (**Fig. 3C**). Of note, the clonogenicity, or ability of a single cell to proliferate and form a colony, is a property commonly associated with cell “stemness”. As expected, satellite cells isolated from uninjured muscle were more clonogenic than those that were activated by injury and had begun to be commit to myogenic differentiation [3] (**Fig. 3C**).

## Discussion

Genome maintenance, and thus DNA damage, is widely considered to be a major culprit in numerous diseases related to aging, as exemplified by many studies showing the rapid progression of age-related symptoms and syndromes in mice with genetic defects in DNA repair pathways [38]. However, the role of DNA damage in the physiological decline of regenerative responses with age in mammals remains undetermined for most tissues.

Skeletal muscle atrophy and decline in regenerative capacity with age has been attributed to an age-related loss of satellite cell functionality. The intrinsic molecular mechanisms underlying this impaired function are poorly understood; one rational hypothesis is that satellite cells experience “intrinsic aging”, rendering them less responsive to environmental cues. This study addressed this possibility with respect to DNA damage and, specifically, its more dramatic lesions, DNA DSBs.

When isolated from uninjured muscle, where the vast majority of muscle stem cells are quiescent, we found no age-specific difference in the percentage of *γ*-H2AX positive satellite cells (**Fig. 1D**); similarly, no age-specific innate accumulation of DNA DSBs was observed in quiescent satellite cells residing in association with uninjured muscle fibers *in vivo* (**Fig. 1G, H**). This is in contrast with publications showing that DNA DSBs accumulate in hematopoietic stem cells with age, leading to their impaired function [9], [10], [12]. Therefore, the role of DNA DSBs in stem cell aging seems to be tissue-dependent and it is unlikely that the aged satellite cells are unable to initiate the activation process due to cell cycle arrest caused by the accumulation of DNA DSBs. In agreement with such a conclusion, aged muscle stem cells are intrinsically capable of efficient myogenesis within hours of youthful modifications of their local niches (e.g., by ectopic activation of Notch-1 [6]).

Recently, reports focusing on cells from tissues with very slow turnover, such as adipocytes and neurons, have shown that post-mitotic terminally differentiated cells are still able to repair DNA DSBs. In particular, it has been shown that both the expression and the activity of DNAPKcs is increased during adipocyte differentiation [39]. Thus, cells with longer life spans and limited regeneration capabilities might emphasize genome integrity. In skeletal muscle, which is a low turnover tissue, the fact that DNA DSBs do not accumulate in satellite cells upon aging may reflect the specifics of a typically quiescent stem cell pool, which has time to repair such damage or does not experience many damaging stimuli.

The number of DNA DSBs increased quickly after activation of both young and old satellite cells by muscle injury. This data suggests that, following activation, satellite cells are more prone to accumulate DNA DSBs either because they are less efficient in DNA repair or because they are more exposed to DNA damaging agents, e.g. ROS [40], [41]. Our data favor the second possibility since satellite cells isolated from injured and uninjured muscle display similar radiosensitivity to gamma radiation (**Fig. 2D, E** **and Fig. S4**); therefore, it is more likely that the increased metabolic state of activated (as opposed to quiescent) satellite cells and/or the high ROS environment of the injured muscle promotes DNA damage.

For actively dividing cells some of the γ-H2AX foci may be generated as a result of stalled DNA replication forks in S phase or single DNA strand breaks [35]. However, a co-localization between 53BP1 and γ-H2AX, the lack of age-specific difference (despite the fact that old cells stall in the cell cycle more than young cells), and detection of the expected difference between wild type and SCID cells all suggest that in our experimental system, DNA DSBs were mostly assayed. Notably, we performed a kinetic experiment on satellite cells isolated from uninjured muscle and injured muscle – 12, 24, and 36 hours after injury – (prior to and after satellite cell entry into the cell cycle [3], [4]) and no age-specific differences were found at any of these time points, which further strengthens our conclusions (**Fig. 1D**).

Satellite cells are equipped to repair DNA DSBs, as shown by the expression of key DNA DSB repair pathway proteins (**Fig. 2A, B**), and they are indeed able to repair DNA DSBs and form myogenic colonies after gamma-radiation (**Fig. 2D, E**). When exposed to gamma-radiation, a slightly higher radiosensitivity was observed in satellite cells isolated from uninjured aged mice, potentially as a consequence of the tendency of DNA DSB repair protein expression to decrease with age (**Fig. 2D**). However, no age-specific difference in radiosensitivity was found among satellite cells that were activated by muscle injury (**Fig. 2E**), which corroborates the findings of the lack of an age-specific difference in the accumulation of innate DNA DSBs in these cells. SCID cells displayed an acute and pronounced radiosensitivity, in perfect agreement with the accumulation of the innate DNA DSBs and the known deficiency in DNA repair (**Fig. 2D, E**). These data suggest that the intrinsic ability of satellite cells isolated from aged mice to repair DNA DSBs does not significantly decline with age.

DNA damage repair deficiency, mutations, and cancer have been strongly associated with one another, as demonstrated in cancer-prone human syndromes such as xeroderma pigmentosum, ataxia-telangiectasia, and Fanconi anemia [14], [38]. Supporting our data that shows a lack of DNA DSB accumulation in muscle stem cells (**Fig. 1**), the occurrence of primary skeletal muscle cancer, or rhabdomyosarcomas, thought to arise from muscle progenitors, is extremely low in adult humans. Additionally, our results are reinforced by data from a previous report which shows that *γ*-H2AX foci are low in skeletal muscle tissue when compared to other tissues and that no increase in *γ*-H2AX foci is observed with age [42].

SCID mice have a defect in DNA repair as a result of a hypomorphic point mutation in DNAPKcs [28]. Despite having more *γ*-H2AX foci and a higher radiosensitivity than found in satellite cells isolated from young or old wild type mice (**Fig. 1B, C** **and** **2D**), SCID satellite cells are robustly myogenic *in vitro* and *in vivo* outperforming satellite cells from old wild type mice (**Fig. 3A–C**). These data demonstrate that a defect in DNA DSB repair does not necessarily correlate with an impaired regenerative performance and that the slight increase in radiosensitivity observed in old satellite cells is unlikely to account for their impaired regenerative capacity.

Comprehensively, these results shed light on the mechanism of stem cell aging, suggesting that the accumulation of DNA DSBs –and perhaps DNA damage in general– is not the key inhibitory culprit of muscle stem cell aging. Our data are in favor of the hypothesis that the muscle stem cell niche plays a key role in the impaired function observed with age as reported in recent studies [3], [43]. Nevertheless, our data does not exclude the possibility that other intrinsic genetic and/or epigenetic changes may contribute to the age-dependent decline in satellite cell myogenicity causing the decline in old muscle repair

## Materials and Methods

### Mice

8-week-old male C57BL/6 mice and 8-week-old male SCID (B6.CB17-Prkdcscid/SzJ) mice were purchased from the Jackson Laboratory. 22-month-old male C57BL/6 mice were purchased from the National Institute on Aging. Animals were housed at the Northwest Animal Facility (University of California, Berkeley) and all procedures were performed in accordance with the administrative panel of the Office of Laboratory Animal Care, UC Berkeley. The protocol was approved by the UC Berkeley Animal Care and Use Committee (ACUC). Mice were anesthetized by isoflurane drop and euthanized by cervical dislocation.

### Satellite Cell Isolation

Tibialis anterior and gastrocnemius muscles of mice were injected with a total of 10 µg of cardiotoxin (Sigma-Aldrich) per leg dissolved in PBS or were left uninjured. Muscles were dissected 3 days post injury and satellite cells were derived as previously described [6], [7]. Briefly, harvested muscles underwent enzymatic digestion in DMEM (Mediatech) containing collagenase type II (250 U/mL; Sigma-Aldrich), 10 mM HEPES, and penicillin/streptomycin dual antibiotic (500 IU/mL, 0.1 mg/mL; MP Biomedicals) at 37°C for 1 hour and 30 minutes under agitation. Fat pads and tendons were removed after a quick wash with PBS and repeated rounds of muscle trituration and sedimentation were performed to purify myofibers. Fibers were then collected after centrifugation and incubated with collagenase type II (40 U/mL) and dispase (2 U/mL; Invitrogen) diluted in Ham's F-10 medium (Mediatech), supplemented with 20% bovine growth serum (Hyclone), and 1% penicillin/streptomycin at 37°C for 1 hour under agitation. Suspensions were vortexed for 1 minute to release satellite cells from digested fibers, passed through a 40 µm cell strainer (BD Biosciences), spun down, and resuspended in growth medium for pre-plating on uncoated dishes for 30 minutes at 37°C, 5% CO2. Cells that did not adhere during pre-plating were collected and plated onto CC2-treated LabTek Chamber Slides (Thermo Fisher Scientific) or 10 cm plates (USA Scientific) coated with 1∶100 Matrigel (BD Biosciences) in growth medium. *In vivo* activated satellite cells were prepared 72 hours post muscle injury and were fixed 12 hours after plating. To avoid *in vitro* activation, satellite cells used in kinetic experiments (**Fig. 1D**) were fixed 4 hours after plating.

### γ-H2AX Immunostaining and Foci Counting Assay

Satellite cells or muscle sections were fixed for 20 minutes at room temperature with 4% paraformaldehyde (PFA) and washed thoroughly with PBS. Cells were permeabilized with 0.25% Triton-X-100 in staining buffer (0.1% Sodium Azide and 1% Bovine Growth Serum in PBS) for 12 minutes and incubated with primary mouse monoclonal anti-*γ*-H2AX antibody (1∶1000) in staining buffer for 2 hours at room temperature. Cells were washed with staining buffer and then incubated with goat-anti-mouse Alexa-Fluor 488 or 546 conjugated secondary antibody (1∶2000) in staining buffer for 2 hours at room temperature. After washing with staining buffer, slides were mounted with ProLong Gold antifade reagent with DAPI (P36935, Invitrogen) and left at room temperature overnight to allow them to dry. Isolated satellite cells were imaged with Zeiss Axio Imager A1 with an Axiocam MRc camera and AxioVision software. Cells were blindly scored for γ-H2AX foci and the number of foci per nuclei was recorded. Foci analysis assay was performed in 3 independent experiments and at least 140 cells per condition were counted for the 72 hours post injury time point. The kinetic was performed on 2 young and 2 old mice per time point and at least 100 cells were counted per mouse. Muscle sections were imaged with the Prairie Technologies Swept Field Confocal scanner on an Olympus BX51W microscope body and an Photometrics QuantEM 512SC camera set on 5 MHz scanning speed with a Olympus 60× oil objective and a Semrock “quad-band” bandpass filter (BrightLine®, FF01-446/523/600/677-25). The number of γ-H2AX positive cells in satellite positions was recorded on Z-stack projected images. A total of 30–50 nuclei were visualized per image. At least 150 nuclei per mice were scored.

### Western Blotting

20 µg of satellite cell nuclear extract protein (each replicate is a pool from 3 mice) per condition was resolved in Laemmli buffer by SDS-PAGE electrophoresis on pre-cast gels (Bio-Rad) and transferred to polyvinylidene fluoride (PVDF) membranes. Membranes were blocked for 30 min in 5% non-fat milk in TBS-0.05% Tween. Primary antibodies against Lamin B1 (1∶10000), Lig4 (1∶500), 53BP1 (1∶1000), NHEJ-1 (1∶300), DNAPK-cs (1∶200), XRCC4 (1∶300), Phospho-ATM (1∶500), Ku70 (1∶500), and Rad51 (1∶1000) were diluted in 5% non-fat milk in TBS-0.05% Tween and antibodies against Rad52 (1∶500) and ATM (1∶1000) were diluted in 5% bovine serum albumin in TBS-0.05% Tween. PVDF membranes were incubated in antibody solutions overnight at 4°C. HRP-conjugated secondary antibodies were diluted 1∶2000 in 5% non-fat milk in TBS-0.05% Tween and incubated with membranes for 2 hours at room temperature. Blots were subsequently developed using Amersham ECL Plus (GE Healthcare), and analyzed with Bio-Rad Gel Doc/Chemi Doc Imaging System and Quantity One Software. Pixel density was analyzed using ImageJ64 software and normalized to LaminB1-specific pixel density.

### Antibodies

Mouse monoclonal antibodies to *γ*-H2AX (05-636) and Rad51 (05-530) were purchased from Millipore. Rabbit polyclonal antibody against Lig4 (12695-1-AP) was purchased from Proteintech Group Inc. Cernunnos-XLF/NHEJ1 (A300-730A) rabbit polyclonal antibody was purchased from Bethyl Laboratories. Rabbit polyclonal antibody against XRCC4 (X4128) was purchased from Sigma-Aldrich. Antibodies to LaminB1 (ab16048, rabbit polyclonal), MyoD (ab3106, mouse monoclonal), Desmin (ab15200, rabbit polyclonal) were purchased from Abcam. Antibodies to Phospho-ATM (4526S, mouse monoclonal), ATM (2873S, rabbit monoclonal), and Rad52 (3425, rabbit polyclonal) were purchased from Cell Signaling Technologies. Mouse monoclonal antibodies against Ku70 (MS-329-P) and DNA-PKcs (MS-423-P1) were purchased from Neomarkers. Antibodies against Pax7 and eMHC were purchased from Developmental Studies Hybridoma Bank. Rabbit polyclonal antibody against 53BP1 (NB100-305) was purchased from Novus Biologicals. Rabbit polyclonal antibody against Myf-5 was purchased from Santa Cruz Biotechnologies (SC-302). Fluorophore-conjugated secondary antibodies (Alexa Fluor) were purchased from Invitrogen. HRP-conjugated secondary antibodies were purchased from Santa Cruz Biotechnologies.

### RNA and qRT-PCR

RNA isolation was performed using RNAeasy Kit (Qiagen) according to manufacturer recommendations. Quantitative RT-PCR was performed using an RT2 First Strand Kit and RT2 Profiler PCR Arrays “DNA Damage Signaling Pathway” PAMM-029 and “DNA Repair” PAMM-042 (SA Biosciences) according to manufacturer recommendations.

### Clonogenic Assay

Freshly-isolated SCID and WT satellite cells from injured and uninjured mice were plated in growth media as single cells on 1∶100 ECM-coated 10 cm tissue culture plates at low density (35 cells per square centimeter). Twelve hours after incubation at 37°C, non-adherent cells were discarded and satellite cells were irradiated at different radiation dosages (0, 1, 2, 3, 4, 5, 10, or 15 Gy) using a ^137^Cs-*γ*-irradiator and media was changed every other day. Ten days after irradiation, cells were washed with cold PBS, fixed and stained for 1 hour with 0.1% Crystal Violet in 2% ethanol and 0.1 M borate [pH 9.0], and washed three times with deionized water. Plates were then scanned and colonies containing at least 50 cells were counted. Prior to fixation and staining, each and every colony from the satellite cell isolation was closely examined under a microscope to determine if its morphology resembled a satellite cell or a different non-myogenic cell lineage. Non-myogenic cell colonies were individually outlined by hand using a pen and were not counted in final tabulations as a surviving myogenic colony from a satellite cell. Each condition (young, old, and SCID) was done using satellite cells isolated from three different mice, with three 10 cm dishes plated per mouse. The result per mouse is a sum of the colonies scored in three dishes and the result per condition is the average of three mice relative to the non-irradiated control +/− SEM. For the non-irradiated control, the number of myogenic colonies (sum of three dishes) ranged between 156 to 175 for young uninjured, 61 to 79 for young injured, 50 to 106 for old uninjured, 30 to 39 for old injured, 219 to 245 for SCID uninjured and 82 to 95 for SCID injured. In irradiated conditions, the number of myogenic colonies decreased due to an expected cell death.

### Muscle histology and immunostaining

We used an injury model consisting of a focal injection of cardiotoxin in the tibialis anterior (TA). Mice were euthanized five days post injury. The TA was dissected and placed in a cold solution of 25% sucrose in PBS for 4 hours. The TA was then quickly rinsed and frozen in Tissue-Tek OCT for cryosectioning. 10 µm cross-sections were obtained and stored at −80°C. Sections were stained with Hematoxylin and Eosin for histology analysis. For immunostaining, sections were fixed for 20 minutes in 4% PFA, permeabilized with 0.25% Triton-X-100 in staining buffer (0.1% Sodium Azide and 1% Bovine Growth Serum in PBS) for 12 minutes, and incubated with primary antibody within staining buffer overnight at 4°C. Sections were washed thoroughly with staining buffer and incubated with an Alexa-Fluor conjugated secondary antibody (1∶2000, Invitrogen) in staining buffer for 2 hours at room temperature. After thorough washing with staining buffer, slides were mounted with ProLong Gold antifade reagent with DAPI (P36935, Invitrogen).

## Supporting Information

Material and Methods S1 Supplementary Materials and Methods.
(DOCX)

Figure S1 **Purity of satellite cell preparation.** (A–B) Activated satellite cells were isolated from muscle of young and old mice 72 hours after injury, fixed and immunostained for Myf-5 or MyoD (green), and counterstained with DAPI (blue). (A) Representative pictures. (B) Quantification of Myf-5 positive and MyoD positive cells. At least 100 cells were counted per mouse. Data represent the mean +/− SEM, n = 3, two-tailed unpaired Student's t-test, no significant difference. Scale bar represents 100 µm. (C) Activated satellite cells were isolated from muscle of young mice 72 hours after muscle injury and immunostained for γ-H2AX (green) and 53BP1 (red) and counterstained with DAPI (blue). Scale bar represents 5 µm.
(TIF)

Figure S2 **No difference in DNA DSB repair signaling pathway gene expression in activated satellite cells from young and old mice.** Graphical representation of data from **Table S1**. Gene expression from old relative to young are presented as fold change with a 95 percent confidence interval for the genes involved in DNA DSB repair, n = 3 mice per group, unpaired Student's t-test, no significant difference. The dotted line represents the young level.
(TIF)

Figure S3 **Morphological discrimination of myogenic colonies.** (A) Colonies were visualized by crystal violet staining. (B) Images of typical myogenic versus non-myogenic colonies by bright field microscopy. Scale bar represents 50 µm. (C) Validation of morphology by immunodetection of Pax7 (red) and desmin (green). DAPI (blue) labels all nuclei. Scale bar represents 100 µm.
(TIF)

Figure S4 **Satellite cells from injured and uninjured muscle display similar radiosensitivity to gamma-radiation.** Satellite cells were freshly isolated from uninjured muscle or 72 hours after muscle injury of C57BL/6 young (A) and old (B) mice. Cells were plated at low density, irradiated at indicated Gray doses and cultured for 10 days. Myogenic colonies formed by irradiated cells were quantified and represented relative to their respective non-irradiated controls. On average, 178 and 67 myogenic colonies were scored per mouse for young uninjured and young injured non-irradiated respectively and 77 and 34 myogenic colonies were scored per mouse for old uninjured and old injured non-irradiated respectively. Data represent the mean +/− SEM, n = 3; no statistical differences were observed using a two-tailed unpaired Student's t-test.
(TIF)

Table S1 **DNA damage and DNA repair signaling pathway gene expression profile in activated satellite cells from young and old mice.** Expression levels of genes involved in DNA damage and repair were quantified using quantitative RT-PCR. Results are presented as fold changes: normalized gene expression (2∧^(− Delta Ct)^) in the test sample divided by the normalized gene expression (2∧^(− Delta Ct)^) in the control sample. Young satellite cells were used as control samples and old satellite cells as test samples. Data were normalized to the geometric mean of internal controls Hprt, Hsp90ab1, Gapdh, and Actin b, n = 3 mice per group, two-tailed unpaired Student's t-test, *p* values<0.05 are indicated in red.
(PDF)
