# Supplementary material for: Regenerative Capacity of Old Muscle Stem Cells Declines without Significant Accumulation of DNA Damage
Source: PLoS One. 2013 May 21;8(5):e63528. doi: 10.1371/journal.pone.0063528 (PMC3660529; doi:10.1371/journal.pone.0063528)
Supplement: Material and Methods S1 — Supplementary Materials and Methods. (DOCX) [file pone.0063528.s001.docx]

**SupPorting MATERIAL AND METHODS**

Immunostaining was performed as described for γ-H2AX. Rabbit polyclonal antibody against 53BP1 (NB100-305) was purchased from Novus Biologicals, rabbit polyclonal antibody against Myf-5 was purchased from Santa Cruz Biotechnologies (SC-302) and antibody to MyoD (ab3106, mouse monoclonal) was purchased from Abcam. MyoD and Myf-5 were incubated overnight at 4ºC. Fluorophore-conjugated secondary antibodies (Alexa Fluor) were purchased from Invitrogen.
