# Supplementary figures and images for: Regenerative Capacity of Old Muscle Stem Cells Declines without Significant Accumulation of DNA Damage
Source: PLoS One. 2013 May 21;8(5):e63528. doi: 10.1371/journal.pone.0063528 (PMC3660529; doi:10.1371/journal.pone.0063528)

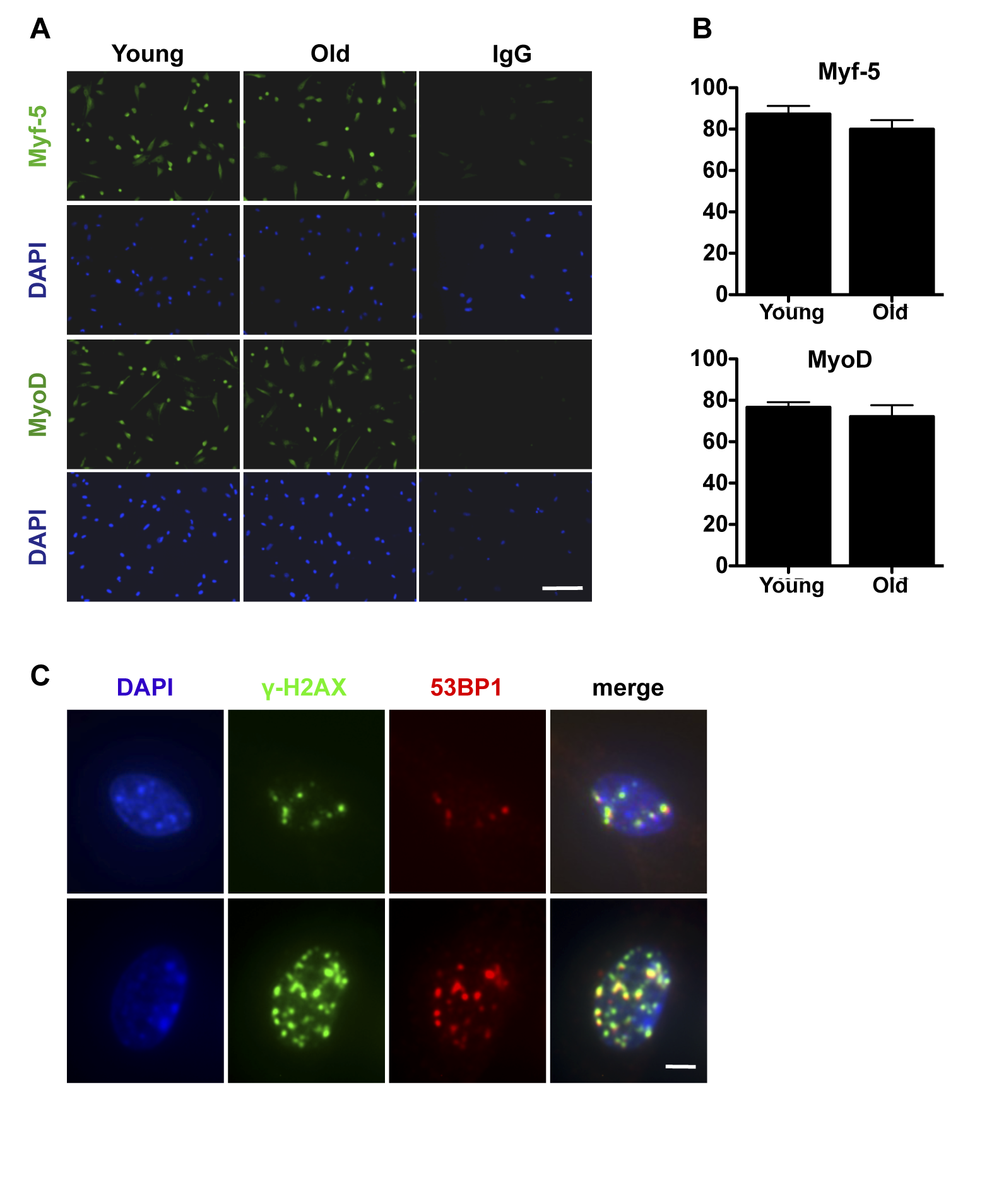

Supplement: Figure S1 — Purity of satellite cell preparation. (A–B) Activated satellite cells were isolated from muscle of young and old mice 72 hours after injury, fixed and immunostained for Myf-5 or MyoD (green), and counterstained with DAPI (blue). (A) Representative pictures. (B) Quantification of Myf-5 positive and MyoD positive cells. At least 100 cells were counted per mouse. Data represent the mean +/− SEM, n = 3, two-tailed unpaired Student's t-test, no significant difference. Scale bar represents 100 µm. (C) Activated satellite cells were isolated from muscle of young mice 72 hours after muscle injury and immunostained for γ-H2AX (green) and 53BP1 (red) and counterstained with DAPI (blue). Scale bar represents 5 µm. (TIF) [file pone.0063528.s002.tif]

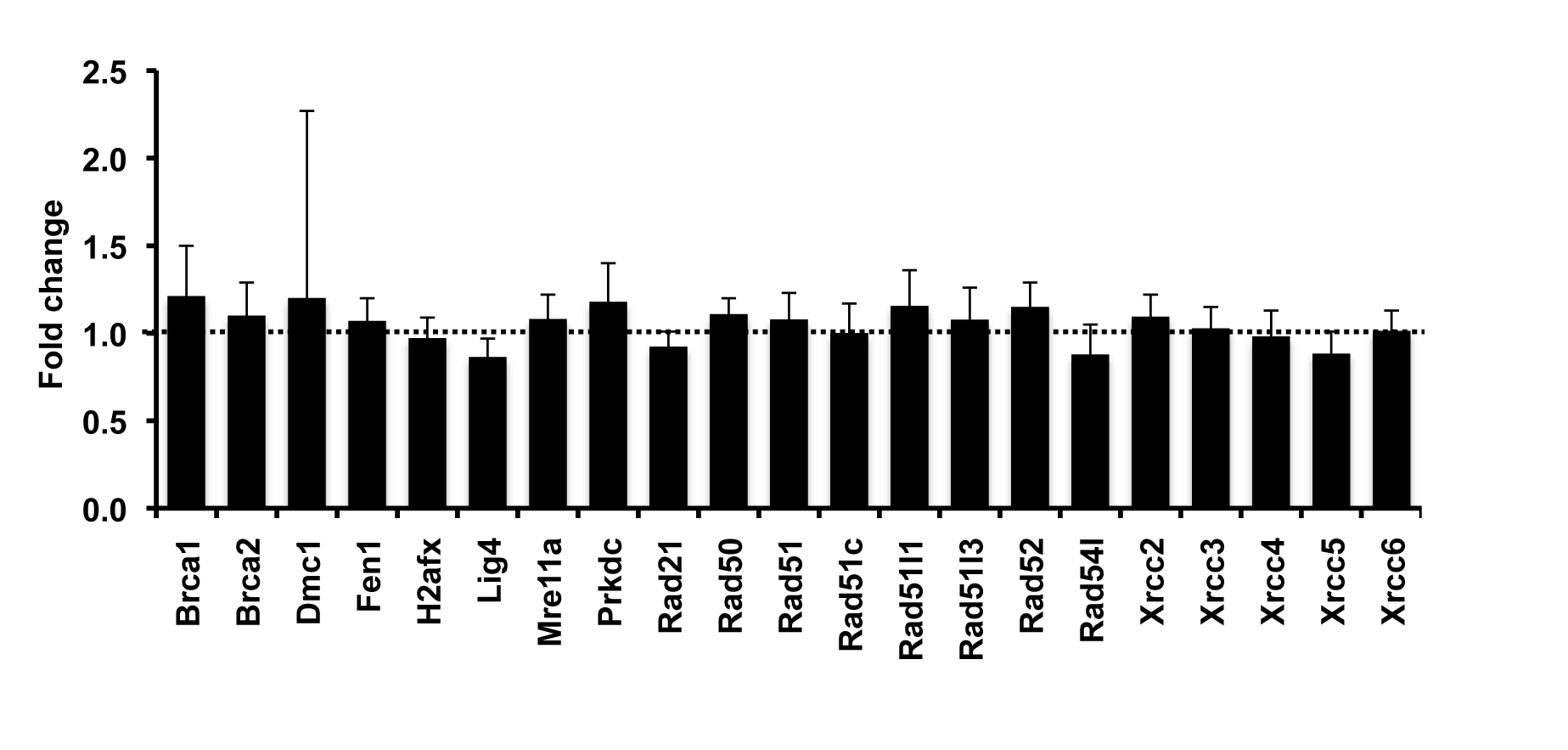

Supplement: Figure S2 — No difference in DNA DSB repair signaling pathway gene expression in activated satellite cells from young and old mice. Graphical representation of data from Table S1. Gene expression from old relative to young are presented as fold change with a 95 percent confidence interval for the genes involved in DNA DSB repair, n = 3 mice per group, unpaired Student's t-test, no significant difference. The dotted line represents the young level. (TIF) [file pone.0063528.s003.tif]

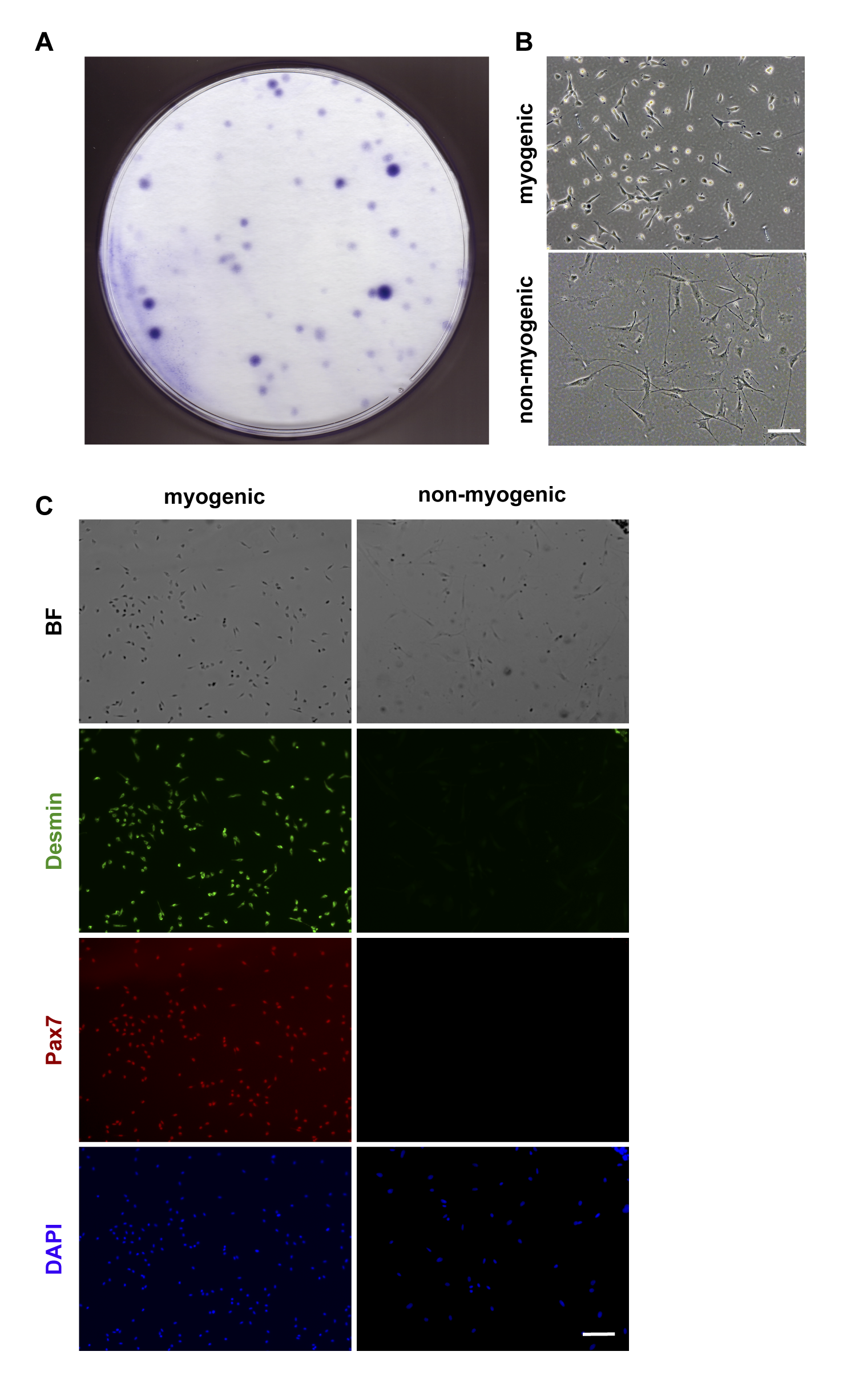

Supplement: Figure S3 — Morphological discrimination of myogenic colonies. (A) Colonies were visualized by crystal violet staining. (B) Images of typical myogenic versus non-myogenic colonies by bright field microscopy. Scale bar represents 50 µm. (C) Validation of morphology by immunodetection of Pax7 (red) and desmin (green). DAPI (blue) labels all nuclei. Scale bar represents 100 µm. (TIF) [file pone.0063528.s004.tif]

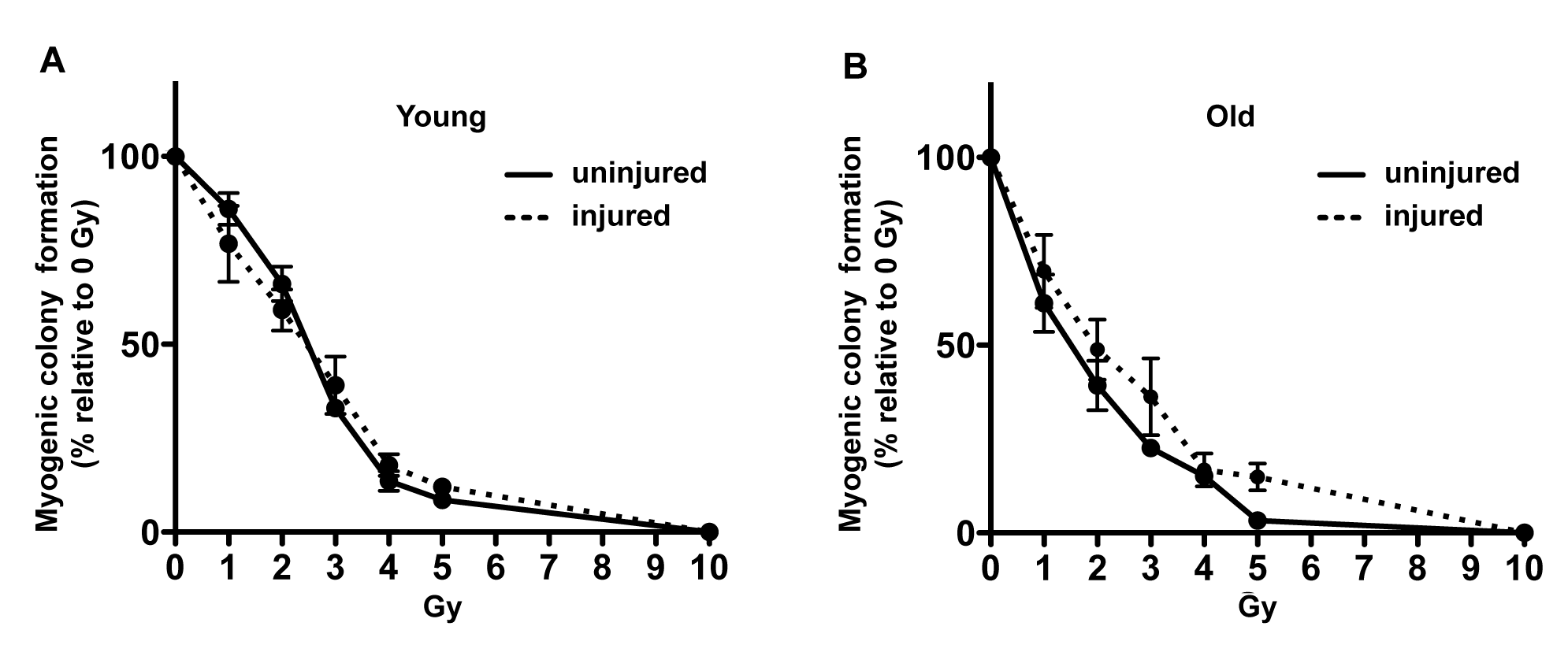

Supplement: Figure S4 — Satellite cells from injured and uninjured muscle display similar radiosensitivity to gamma-radiation. Satellite cells were freshly isolated from uninjured muscle or 72 hours after muscle injury of C57BL/6 young (A) and old (B) mice. Cells were plated at low density, irradiated at indicated Gray doses and cultured for 10 days. Myogenic colonies formed by irradiated cells were quantified and represented relative to their respective non-irradiated controls. On average, 178 and 67 myogenic colonies were scored per mouse for young uninjured and young injured non-irradiated respectively and 77 and 34 myogenic colonies were scored per mouse for old uninjured and old injured non-irradiated respectively. Data represent the mean +/− SEM, n = 3; no statistical differences were observed using a two-tailed unpaired Student's t-test. (TIF) [file pone.0063528.s005.tif]
