# Supplementary material for: Regenerative Capacity of Old Muscle Stem Cells Declines without Significant Accumulation of DNA Damage
Source: PLoS One. 2013 May 21;8(5):e63528. doi: 10.1371/journal.pone.0063528 (PMC3660529; doi:10.1371/journal.pone.0063528)
Supplement: Table S1 — DNA damage and DNA repair signaling pathway gene expression profile in activated satellite cells from young and old mice. Expression levels of genes involved in DNA damage and repair were quantified using quantitative RT-PCR. Results are presented as fold changes: normalized gene expression (2∧(− Delta Ct)) in the test sample divided by the normalized gene expression (2∧(− Delta Ct)) in the control sample. Young satellite cells were used as control samples and old satellite cells as test samples. Data were normalized to the geometric mean of internal controls Hprt, Hsp90ab1, Gapdh, and Actin b, n = 3 mice per group, two-tailed unpaired Student's t-test, p values<0.05 are indicated in red. (PDF) [file pone.0063528.s006.pdf]

Table S1

	Symbol	Refseq	Old vs young	
			Fold Change	p-value
**Apoptosis**	Atm	NM_007499	1.14	0.2595
	Brca1	NM_009764	1.21	0.2001
	Mbd4	NM_010774	1.17	0.0576
	Mgmt	NM_008598	1.03	0.4526
	Mlh1	NM_026810	1.11	0.0717
	Prkdc	NM_011159	1.18	0.1651
	Rad21	NM_009009	0.92	0.1796
	Trp53	NM_011640	0.99	0.7870
**Cell Cycle Arrest**	Chek1	NM_007691	1.04	0.4769
	Gadd45a	NM_007836	0.85	0.0673
	Hus1	NM_008316	1.04	0.4726
	Msh2	NM_008628	1.03	0.2917
**Cell Cycle Checkpoint**	Brca2	NM_009765	1.10	0.3299
	Pinx1	NM_028228	1.04	0.5702
	Rad1	NM_011232	1.00	0.9954
	Rad9	NM_011237	1.04	0.7445
	Smc1a	NM_019710	1.09	0.1296
**Cell Cycle Related**	Atm	NM_007499	1.14	0.2595
	Chaf1a	NM_013733	1.03	0.8364
	Rad21	NM_009009	0.92	0.1796
	Rbbp4	NM_009030	0.94	0.4231
	Smc3	NM_007790	0.99	0.8595
	Terf1	NM_009352	1.09	0.3338
	Tlk1	NM_172664	1.04	0.4788
**Double-Strand Break repair**	Brca1	NM_009764	1.21	0.2001
	Brca2	NM_009765	1.10	0.3299
	Dmc1	NM_010059	1.20	0.5257
	Fen1	NM_007999	1.07	0.3573
	H2afx	NM_010436	0.97	0.6551
	Lig4	NM_176953	0.86	0.0768
	Mre11a	NM_018736	1.08	0.3193
	Prkdc	NM_011159	1.18	0.1651
	Rad21	NM_009009	0.92	0.1796
	Rad50	NM_009012	1.11	0.0594
	Rad51	NM_011234	1.08	0.3403
	Rad51c	NM_053269	1.00	0.9577
	Rad51l1	NM_009014	1.16	0.1862
	Rad51l3	NM_011235	1.08	0.4290

	Rad52	NM_011236	1.15	0.0933
	Rad54l	NM_009015	0.88	0.2520
	Xrcc2	NM_020570	1.09	0.1849
	Xrcc3	NM_028875	1.03	0.7082
	Xrcc4	NM_028012	0.98	0.8000
	Xrcc5	NM_009533	0.88	0.1674
	Xrcc6	NM_010247	1.01	0.7755
**Damaged DNA Binding**	Brca1	NM_009764	1.21	0.2001
	Ercc1	NM_007948	0.96	0.5727
	H2afx	NM_010436	0.97	0.6551
	Msh2	NM_008628	1.03	0.2917
	Msh3	NM_010829	1.06	0.2798
	Rad1	NM_011232	1.00	0.9954
	Rad51	NM_011234	1.08	0.3403
	Rad51c	NM_053269	1.00	0.9577
	Rad51l1	NM_009014	1.16	0.1862
	Trpc2	NM_011644	1.24	0.1598
	Xpa	NM_011728	1.10	0.1260
	Xpc	NM_009531	0.96	0.7057
	Xrcc1	NM_009532	0.89	0.0354
	Xrcc2	NM_020570	1.09	0.1849
	Xrcc3	NM_028875	1.03	0.7082
**Base Excision Repair**	Apex1	NM_009687	1.05	0.5033
	Apex2	NM_029943	1.13	0.0219
	Ccno	NM_001081062	1.37	0.1940
	Lig3	NM_010716	1.03	0.6610
	Mbd4	NM_010774	1.17	0.0576
	Mpg	NM_010822	1.02	0.5221
	Mutyh	NM_133250	1.08	0.2340
	Neil1	NM_028347	0.96	0.5825
	Neil2	NM_201610	1.14	0.6499
	Neil3	NM_146208	0.86	0.1348
	Nthl1	NM_008743	1.04	0.7094
	Ogg1	NM_010957	1.04	0.6372
	Parp1	NM_007415	0.99	0.7098
	Parp2	NM_009632	1.14	0.1927
	Parp3	NM_145619	1.28	0.3461
	Polb	NM_011130	0.86	0.0306
	Smug1	NM_027885	0.95	0.4557
	Tdg	NM_011561	0.95	0.5535
	Ung	NM_011677	1.06	0.4679
	Xrcc1	NM_009532	0.89	0.0354
**Nucleotide-excision Repair**	Atxn3	NM_029705	0.95	0.3230

	Brip1	NM_178309	1.03	0.6550
	Ccnh	NM_023243	0.98	0.7194
	Cdk7	NM_009874	0.80	0.1612
	Dclre1a	NM_018831	1.23	0.1280
	Ddb1	NM_015735	0.85	0.0348
	Ddb2	NM_028119	1.13	0.2722
	Ercc1	NM_007948	0.96	0.5727
	Ercc2	NM_007949	1.04	0.5775
	Ercc3	NM_133658	0.89	0.2093
	Ercc4	NM_015769	1.11	0.1298
	Ercc5	NM_011729	0.90	0.1929
	Ercc6	NM_001081221	0.72	0.2485
	Ercc8	NM_028042	0.92	0.6078
	Fancc	NM_007985	1.10	0.2320
	Lig1	NM_010715	1.02	0.4081
	Mms19	NM_028152	0.97	0.6422
	Nthl1	NM_008743	1.04	0.7094
	Pnkp	NM_021549	0.91	0.1243
	Poll	NM_020032	0.92	0.2330
	Rad23a	NM_009010	1.03	0.6359
	Rad23b	NM_009011	1.15	0.2510
	Rpa1	NM_026653	1.05	0.4996
	Rpa3	NM_026632	0.93	0.2431
	Slk	NM_009289	1.08	0.0424
	Xab2	NM_026156	1.01	0.8596
	Xpa	NM_011728	1.10	0.1260
	Xpc	NM_009531	0.96	0.7057
**Mismatch Repair**	Mlh1	NM_026810	1.11	0.0717
	Mlh3	NM_175337	1.15	0.0109
	Msh2	NM_008628	1.03	0.2917
	Msh3	NM_010829	1.06	0.2798
	Msh4	NM_031870	1.50	0.2275
	Msh5	NM_013600	0.91	0.2441
	Msh6	NM_010830	0.90	0.1180
	Pms1	NM_153556	1.08	0.1300
	Pms2	NM_008886	1.21	0.0202
	Pold3	NM_133692	1.14	0.0164
	Trex1	NM_011637	1.42	0.0236
**DNA repair Related**	Atm	NM_007499	1.14	0.2595
	Atr	NM_019864	0.85	0.3628
	Atrx	NM_009530	1.13	0.3379
	Chaf1a	NM_013733	1.03	0.8364
	Cry2	NM_009963	1.09	0.2835

	Exo1	NM_012012	0.58	0.3763
	Fancg	NM_053081	1.00	0.9358
	Fen1	NM_007999	1.07	0.3573
	Gtf2h1	NM_008186	1.09	0.1117
	Gtf2h2	NM_022011	1.06	0.3269
	Lig1	NM_010715	1.02	0.4081
	Mgmt	NM_008598	1.03	0.4526
	Mif	NM_010798	1.03	0.6745
	Pold1	NM_011131	1.10	0.3742
	Pole	NM_011132	1.04	0.5916
	Polh	NM_030715	1.01	0.5991
	Poli	NM_011972	1.06	0.5399
	Polk	NM_012048	1.15	0.1116
	Pttg1	NM_013917	1.64	0.0109
	Rad18	NM_021385	1.06	0.3365
	Rad21	NM_009009	0.92	0.1796
	Rad50	NM_009012	1.11	0.0594
	Rad9	NM_011237	1.04	0.7445
	Rad9b	NM_144912	1.21	0.1261
	Rbbp4	NM_009030	0.94	0.4231
	Rbm4	NM_009032	0.94	0.1597
	Rev1	NM_019570	1.11	0.3661
	Rfc1	NM_011258	0.98	0.7513
	Smc1a	NM_019710	1.09	0.1296
	Smc3	NM_007790	0.99	0.8595
	Srd5a2	NM_053188	0.90	0.4235
	Sumo1	NM_009460	1.07	0.1292
	Tdg	NM_011561	0.95	0.5535
	Top3a	NM_009410	2.35	0.3662
	Top3b	NM_011624	0.92	0.2535
	Ube2a	NM_019668	1.08	0.3856
	Ung	NM_011677	1.06	0.4679
	Wrn	NM_011721	1.15	0.0140
	Wrnip1	NM_030215	1.03	0.7933
	Xrcc6bp1	NM_026858	0.86	0.3459
	Xrn2	NM_011917	1.04	0.5769
